# *N*-(4-Chloro­benzyl­idene)-4-methoxy­aniline

**DOI:** 10.1107/S1600536808026111

**Published:** 2008-08-20

**Authors:** Xiao-Yan Ren, Yu-Feng Ding, Fang-Fang Jian

**Affiliations:** a Microscale Science Institute, Weifang University, Weifang 261061, People’s Republic of China; b The 7th Middle School, Weifang 261061, People’s Republic of China

## Abstract

The title compound, C_14_H_12_ClNO, was prepared by the reaction of 4-methoxy­aniline and 4-chloro­benzaldehyde in ethanol at 367 K. The mol­ecule is almost planar, with a dihedral angle between the two benzene rings of 9.1 (2)° and an r.m.s. deviation from the mean plane through all non-H atoms in the mol­ecule of 0.167 Å.

## Related literature

For applications of Schiff base compounds, see: Deschamps *et al.* (2003 ▶); Rozwadowski *et al.* (1999 ▶); Tarafder *et al.* (2000 ▶). For a related structure, see: Jian *et al.* (2006 ▶).
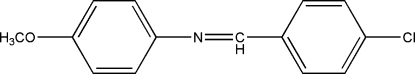


## Experimental

### 

#### Crystal data

C_14_H_12_ClNO
*M* *_r_* = 245.70
Orthorhombic, 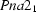

*a* = 6.1055 (9) Å
*b* = 7.3392 (11) Å
*c* = 27.469 (4) Å
*V* = 1230.9 (3) Å^3^
*Z* = 4
Mo *K*α radiation
μ = 0.29 mm^−1^
*T* = 293 (2) K
0.20 × 0.15 × 0.11 mm

#### Data collection

Bruker SMART CCD area-detector diffractometer
Absorption correction: none
7232 measured reflections
2806 independent reflections
2092 reflections with *I* > 2σ(*I*)
*R*_int_ = 0.021

#### Refinement

*R*[*F* ^2^ > 2σ(*F* ^2^)] = 0.035
*wR*(*F* ^2^) = 0.088
*S* = 1.01
2806 reflections
154 parameters
1 restraint
H-atom parameters constrained
Δρ_max_ = 0.14 e Å^−3^
Δρ_min_ = −0.16 e Å^−3^
Absolute structure: Flack (1983 ▶), 1450 Friedel pairs
Flack parameter: −0.01 (7)

### 

Data collection: *SMART* (Bruker, 1997 ▶); cell refinement: *SAINT* (Bruker, 1997 ▶); data reduction: *SAINT*; program(s) used to solve structure: *SHELXS97* (Sheldrick, 2008 ▶); program(s) used to refine structure: *SHELXL97* (Sheldrick, 2008 ▶); molecular graphics: *SHELXTL* (Sheldrick, 2008 ▶); software used to prepare material for publication: *SHELXTL*.

## Supplementary Material

Crystal structure: contains datablocks global, I. DOI: 10.1107/S1600536808026111/sj2527sup1.cif

Structure factors: contains datablocks I. DOI: 10.1107/S1600536808026111/sj2527Isup2.hkl

Additional supplementary materials:  crystallographic information; 3D view; checkCIF report

## Figures and Tables

**Fig. 1.**
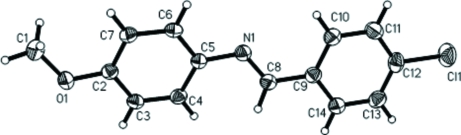
The structure of the title compound showing 30% probability displacement ellipsoids and the atom-numbering scheme.

## supplementary crystallographic information

### Comment

Schiff bases have been used extensively as ligands in the field of coordination chemistry (Jian *et al.*, 2006), and have antimicrobial (Tarafder *et al.*, 2000) and anticancer applications (Deschamps *et al.*, 2003). Additional recent interest in Schiff base compounds comes from their ability to form intramolecular hydrogen bonds by electron coupling between acid-base centers (Rozwadowski *et al.*,1999).  We report here the synthesis and structure of the title Schiff base compound, I, Fig. 1.

The molecule is almost planar with a dihedral angle between the C2···C7 and C9···C13 benzene rings  of  9.1 (2)° and an rms deviation from the meanplane through all non-hydrogen atoms in the molecule of 0.167.  The C═N bond distance (1.255 (2) Å) is in reasonable agreement with that observed in a similar compound (Jian *et al.*, 2006).

### Experimental

A mixture of 4-methoxyaniline 2.46 g (0.02 mol) and 4-chlorobenzaldehyde 2.8 g (0.02 mol) was stirred in ethanol (50 mL) at 367 K for 2 h, to give the title compound (3.9 g, yield 81%). Single crystals suitable for X-ray measurements were obtained by recrystallization from acetone and ethanol(1:1) at room temperature.

### Refinement

All H-atoms were positioned geometrically and refined using a riding model with d(C-H) = 0.93Å,  U_iso_=1.2U_eq_(C) for aromatic and 0.96Å,  U_iso_ = 1.5U_eq_(C) for CH_3_ atoms.

### Crystal data

**Table:**

C_14_H_12_ClNO	*F*_000_ = 512
*M**_r_* = 245.70	*D*_x_ = 1.326 Mg m^−^^3^
Orthorhombic, *P**n**a*2_1_	Mo *K*α radiation λ = 0.71073 Å
Hall symbol: P 2c -2n	Cell parameters from 2092 reflections
*a* = 6.1055 (9) Å	θ = 2.9–28.3º
*b* = 7.3392 (11) Å	µ = 0.29 mm^−^^1^
*c* = 27.469 (4) Å	*T* = 293 (2) K
*V* = 1230.9 (3) Å^3^	Block, yellow
*Z* = 4	0.20 × 0.15 × 0.11 mm

### Data collection

**Table:**

Bruker SMART CCD area-detector diffractometer	2092 reflections with *I* > 2σ(*I*)
Radiation source: fine-focus sealed tube	*R*_int_ = 0.022
Monochromator: graphite	θ_max_ = 28.3º
*T* = 293(2) K	θ_min_ = 2.9º
φ and ω scans	*h* = −8→4
Absorption correction: none	*k* = −9→9
7232 measured reflections	*l* = −36→33
2806 independent reflections	

### Refinement

**Table:**

Refinement on *F*^2^	Hydrogen site location: inferred from neighbouring sites
Least-squares matrix: full	H-atom parameters constrained
*R*[*F*^2^ > 2σ(*F*^2^)] = 0.035	*w* = 1/[σ^2^(*F*_o_^2^) + (0.04*P*)^2^ + 0.0836*P*] where *P* = (*F*_o_^2^ + 2*F*_c_^2^)/3
*wR*(*F*^2^) = 0.088	(Δ/σ)_max_ < 0.001
*S* = 1.01	Δρ_max_ = 0.14 e Å^−^^3^
2806 reflections	Δρ_min_ = −0.16 e Å^−^^3^
154 parameters	Extinction correction: none
1 restraint	Absolute structure: Flack (1983), 1450 Friedel pairs
Primary atom site location: structure-invariant direct methods	Flack parameter: −0.01 (7)
Secondary atom site location: difference Fourier map	

### Special details

**Table:**

Geometry. All e.s.d.'s (except the e.s.d. in the dihedral angle between two l.s. planes) are estimated using the full covariance matrix. The cell e.s.d.'s are taken into account individually in the estimation of e.s.d.'s in distances, angles and torsion angles; correlations between e.s.d.'s in cell parameters are only used when they are defined by crystal symmetry. An approximate (isotropic) treatment of cell e.s.d.'s is used for estimating e.s.d.'s involving l.s. planes.
Refinement. Refinement of *F*^2^ against ALL reflections. The weighted *R*-factor *wR* and goodness of fit *S* are based on *F*^2^, conventional *R*-factors *R* are based on *F*, with *F* set to zero for negative *F*^2^. The threshold expression of *F*^2^ > σ(*F*^2^) is used only for calculating *R*-factors(gt) *etc*. and is not relevant to the choice of reflections for refinement. *R*-factors based on *F*^2^ are statistically about twice as large as those based on *F*, and *R*- factors based on ALL data will be even larger.

### Fractional atomic coordinates and isotropic or equivalent isotropic displacement parameters (Å^2^)

**Table:**

	*x*	*y*	*z*	*U*_iso_*/*U*_eq_	
Cl1	0.77772 (12)	0.00540 (10)	0.23599 (3)	0.0862 (2)	
O1	−0.0611 (2)	−0.01368 (19)	0.64134 (5)	0.0560 (4)	
N1	0.2457 (3)	0.0122 (2)	0.45058 (6)	0.0463 (4)	
C1	−0.2589 (4)	0.0731 (4)	0.65469 (10)	0.0750 (7)	
H1B	−0.2859	0.0539	0.6887	0.112*	
H1C	−0.3775	0.0230	0.6361	0.112*	
H1D	−0.2475	0.2014	0.6483	0.112*	
C2	0.0048 (3)	−0.0027 (2)	0.59389 (7)	0.0421 (4)	
C3	0.1994 (3)	−0.0915 (2)	0.58341 (7)	0.0446 (4)	
H3A	0.2739	−0.1522	0.6081	0.053*	
C4	0.2838 (3)	−0.0909 (3)	0.53699 (7)	0.0456 (4)	
H4A	0.4144	−0.1513	0.5305	0.055*	
C5	0.1737 (3)	0.0005 (2)	0.49944 (7)	0.0395 (4)	
C6	−0.0225 (3)	0.0856 (3)	0.51062 (6)	0.0436 (4)	
H6A	−0.0996	0.1442	0.4860	0.052*	
C7	−0.1074 (3)	0.0862 (2)	0.55747 (7)	0.0451 (4)	
H7A	−0.2384	0.1458	0.5642	0.054*	
C8	0.4424 (3)	−0.0157 (2)	0.43961 (7)	0.0475 (5)	
H8A	0.5407	−0.0439	0.4644	0.057*	
C9	0.5235 (3)	−0.0060 (2)	0.38958 (8)	0.0456 (4)	
C10	0.3951 (4)	0.0631 (3)	0.35228 (7)	0.0537 (5)	
H10A	0.2559	0.1075	0.3592	0.064*	
C11	0.4712 (4)	0.0666 (3)	0.30525 (8)	0.0595 (6)	
H11A	0.3842	0.1133	0.2804	0.071*	
C12	0.6790 (4)	0.0000 (3)	0.29508 (8)	0.0572 (6)	
C13	0.8091 (3)	−0.0666 (3)	0.33165 (8)	0.0579 (5)	
H13A	0.9480	−0.1115	0.3247	0.069*	
C14	0.7321 (3)	−0.0665 (3)	0.37880 (7)	0.0534 (5)	
H14A	0.8221	−0.1079	0.4038	0.064*	

### Atomic displacement parameters (Å^2^)

**Table:**

	*U*^11^	*U*^22^	*U*^33^	*U*^12^	*U*^13^	*U*^23^
Cl1	0.0943 (5)	0.1126 (5)	0.0517 (3)	−0.0069 (4)	0.0194 (4)	−0.0127 (3)
O1	0.0570 (9)	0.0702 (10)	0.0409 (7)	0.0069 (7)	0.0025 (7)	−0.0003 (6)
N1	0.0453 (10)	0.0487 (10)	0.0448 (10)	0.0027 (7)	−0.0005 (7)	−0.0006 (7)
C1	0.0614 (15)	0.104 (2)	0.0598 (14)	0.0089 (13)	0.0097 (11)	−0.0055 (13)
C2	0.0414 (9)	0.0408 (9)	0.0440 (10)	−0.0062 (7)	−0.0034 (9)	−0.0031 (8)
C3	0.0429 (9)	0.0450 (10)	0.0459 (11)	0.0016 (8)	−0.0062 (8)	0.0036 (8)
C4	0.0396 (10)	0.0457 (10)	0.0516 (11)	0.0073 (8)	−0.0017 (8)	0.0024 (8)
C5	0.0408 (10)	0.0361 (9)	0.0417 (9)	−0.0022 (7)	−0.0009 (7)	−0.0013 (7)
C6	0.0409 (10)	0.0411 (10)	0.0487 (11)	0.0043 (8)	−0.0080 (8)	0.0020 (8)
C7	0.0397 (10)	0.0444 (9)	0.0511 (11)	0.0048 (8)	−0.0023 (8)	−0.0014 (8)
C8	0.0453 (11)	0.0527 (11)	0.0446 (10)	−0.0005 (9)	−0.0036 (9)	−0.0018 (9)
C9	0.0435 (10)	0.0449 (10)	0.0484 (10)	−0.0041 (8)	−0.0011 (9)	−0.0025 (8)
C10	0.0479 (11)	0.0593 (12)	0.0540 (12)	0.0032 (10)	0.0019 (9)	−0.0013 (9)
C11	0.0598 (14)	0.0688 (14)	0.0498 (12)	0.0060 (11)	−0.0051 (10)	0.0013 (9)
C12	0.0660 (14)	0.0581 (13)	0.0476 (12)	−0.0085 (11)	0.0123 (10)	−0.0092 (10)
C13	0.0473 (12)	0.0646 (13)	0.0617 (14)	0.0032 (10)	0.0059 (10)	−0.0055 (11)
C14	0.0473 (12)	0.0620 (13)	0.0508 (12)	0.0043 (9)	−0.0002 (9)	0.0018 (9)

### Geometric parameters (Å, °)

**Table:**

Cl1—C12	1.732 (2)	C6—C7	1.387 (2)
O1—C2	1.366 (2)	C6—H6A	0.9300
O1—C1	1.414 (3)	C7—H7A	0.9300
N1—C8	1.255 (2)	C8—C9	1.463 (3)
N1—C5	1.415 (3)	C8—H8A	0.9300
C1—H1B	0.9600	C9—C14	1.381 (3)
C1—H1C	0.9600	C9—C10	1.386 (3)
C1—H1D	0.9600	C10—C11	1.373 (3)
C2—C7	1.377 (3)	C10—H10A	0.9300
C2—C3	1.386 (2)	C11—C12	1.388 (3)
C3—C4	1.375 (2)	C11—H11A	0.9300
C3—H3A	0.9300	C12—C13	1.371 (3)
C4—C5	1.402 (3)	C13—C14	1.378 (3)
C4—H4A	0.9300	C13—H13A	0.9300
C5—C6	1.386 (3)	C14—H14A	0.9300
			
C2—O1—C1	118.19 (17)	C2—C7—H7A	120.4
C8—N1—C5	121.00 (17)	C6—C7—H7A	120.4
O1—C1—H1B	109.5	N1—C8—C9	122.78 (18)
O1—C1—H1C	109.5	N1—C8—H8A	118.6
H1B—C1—H1C	109.5	C9—C8—H8A	118.6
O1—C1—H1D	109.5	C14—C9—C10	118.7 (2)
H1B—C1—H1D	109.5	C14—C9—C8	119.87 (19)
H1C—C1—H1D	109.5	C10—C9—C8	121.40 (17)
O1—C2—C7	125.07 (16)	C11—C10—C9	120.7 (2)
O1—C2—C3	115.02 (17)	C11—C10—H10A	119.6
C7—C2—C3	119.91 (18)	C9—C10—H10A	119.6
C4—C3—C2	120.82 (17)	C10—C11—C12	119.5 (2)
C4—C3—H3A	119.6	C10—C11—H11A	120.3
C2—C3—H3A	119.6	C12—C11—H11A	120.3
C3—C4—C5	120.27 (17)	C13—C12—C11	120.5 (2)
C3—C4—H4A	119.9	C13—C12—Cl1	119.55 (17)
C5—C4—H4A	119.9	C11—C12—Cl1	119.92 (19)
C6—C5—C4	117.86 (18)	C12—C13—C14	119.42 (18)
C6—C5—N1	116.83 (16)	C12—C13—H13A	120.3
C4—C5—N1	125.31 (16)	C14—C13—H13A	120.3
C5—C6—C7	122.00 (17)	C13—C14—C9	121.1 (2)
C5—C6—H6A	119.0	C13—C14—H14A	119.5
C7—C6—H6A	119.0	C9—C14—H14A	119.5
C2—C7—C6	119.11 (17)		
